# Putting the “Love of Humanity” Back in Corporate Philanthropy: The Case of Health Grants by Corporate Foundations

**DOI:** 10.1007/s10551-021-04807-2

**Published:** 2021-04-10

**Authors:** Muhammad Umar Boodoo, Irene Henriques, Bryan W. Husted

**Affiliations:** 1 Warwick Business School, University of Warwick, WBS 2.135, Scarman Road, Coventry, CV4 7AL UK; 2 Schulich School of Business, York University, 4700 Keele Street, Toronto, ON M3J 1P3 Canada; 3 EGADE Business School, Tecnológico de Monterrey, Av. Eugenio Garza Laguera y Rufno Tamayo, 66269 San Pedro Garza García, N.L. México

**Keywords:** Corporate philanthropy, Health inequality, Corporate social responsibility (CSR), Homophily, Health grants

## Abstract

With the growing call for private sector actors to address global challenges, it is necessary to first assess whether regions with the greatest needs are accessing corporate philanthropy. In this paper, we ask whether corporate philanthropy is reaching those with the greatest health-care needs. Drawing on economic geography and corporate homophily, we argue that corporate philanthropy tends to exacerbate health inequality as grants are destined for counties with fewer health problems. We test and find support for this hypothesis using data on health grants made by US corporate foundations and county-level health data. Our results that corporate health grants are less likely to go to counties which have a lower proportion of medical service providers and insured citizens suggest that corporate foundations are unwittingly complicit in worsening the resource gap between small, poor, rural counties and large, wealthy, urban counties. From an ethical perspective, we provide some guidance as to how this may be corrected.

## Introduction

The COVID-19 pandemic has exposed the inequalities of the U.S. health-care system. Anecdotal evidence in New York City suggests that at least one public hospital’s intensive care units were required to use flimsy tarps and duct tape to separate patients, while wealthy private hospitals were able to use reserves and political clout to ramp up capacity, testing and acquire protective equipment. The embedded inequalities appear to have been exacerbated by corporate philanthropy. While doctors and nurses in public hospitals were obliged to create GoFundMe pages to raise money for protective gear, “the Mount Sinai health system was able to enlist private planes from Warren E. Buffett’s company to fly in coveted N95 masks from China” (Schwirtz, 2020).

According to (Reich, 2005, p. 26) the “primary motivation for charity has always been to provide for the poor and disadvantaged, and to attack the root causes of poverty and disadvantage.” Americans in 2017 donated $410 billion to charities of which 9% were health-related and 21% of these donations were by foundations and corporations (Nonprofits Source, 2021). The World Health Organization defines health inequality as the difference in health status or the distribution of health determinants between different population groups such as between people from different income groups or social classes (World Health Organization, 2017). The question we seek to address is: Does corporate philanthropy go to regions with the greatest health-care needs?

From a purely rational view, one would expect that a corporate foundation’s philanthropic grant-making for health would be based on the needs of the recipient community. However, there exists significant concern that communities with higher needs, but fewer resources are often handicapped in competitive calls for proposals for health grants (Cantrell et al., 2008; Murday & Corley, 2008).

We argue that two related institutional conditions—homophily and proximity—disadvantage communities with greater needs. Homophily captures the idea that people with certain characteristics tend to associate with others with similar characteristics and is illustrated by the well-known aphorism, “birds of a feather flock together” (McPherson et al., 2001). Proximity refers to the nearness or closeness of things in space, time, or attributes (Boschma, 2005). Generally, we will focus on geographic proximity, unless specified otherwise. Homophily and proximity work to harm dissimilar and distant communities in two ways. First, homophily, or the lack thereof, between corporate foundations and recipient communities influences the choice of recipients (Kallman, 2017; Spires, 2011), and the proximity of beneficiaries with respect to corporate operations and their foundations (Muller & Whiteman, 2016) alters the costs and benefits by reducing the costs of information search, and thus affects the target and amounts of foundation grants to beneficiaries. Second, homophily and proximity affect the institutions and legitimating pressures to which corporate foundations are subject, thus affecting their giving behavior (Galaskiewicz, 1997; Marquis et al., 2007). In summary, homophily and proximity influence the incentives and constraints set by different institutional arrangements, thus affecting health equity.

We explore corporate philanthropy and its relationship to health inequality in the context of health grants made by US corporate foundations to determine whether these grants targeted the counties with greatest needs or not. By health needs we refer to “the capacity to benefit” from effective interventions and available resources (Wright et al., 1998, p. 1311). We examine the question by building a unique dataset drawn from data on grants by corporate foundations from Candid (formerly the Foundation Center), and data on health outcomes compiled for the County Health Rankings, a program of the Robert Wood Johnson Foundation. First, we find significant differences in characteristics among counties, such that there is a negative relationship between health needs and the likelihood of grants received. Furthermore, contingent on receiving donations, counties with greater health needs are associated with fewer health grants per capita. Since this is an observational study and not a randomized trial, causality cannot be established but a strong association is found. If causality were to be established, then grants from corporate foundations would be reinforcing pre-existing health inequalities such that needy counties, that is, those which have more uninsured citizens and where citizens have lower access to primary-care physicians and mental health providers, are less likely to be recipients of health-related grants from corporate foundations. We also find significant differences in U.S counties that receive corporate health grants, with recipient counties being more urban than counties that do not receive such grants. If a county is home to the headquarters of at least one corporation which has a foundation, then its likelihood of receiving grants and the amount it receives are both significantly higher.

Our findings shed light on the mixed results of prior research, which have not used a rigorous approach to compare the awarding of grants to urban versus rural counties (Ashley, 2014). Health researchers have found that rural Americans face persistent health disparities compared to people living in urban areas (Miller & Vasan, 2021) because rural Americans tend to have less access to health-care and experience high rates of disease and death (Cosby et al., 2018). Our findings that corporate health-giving targets urban counties suggest that corporate giving may exacerbate such disparities. Given the latter, firms and their foundations need to change the criteria they employ in awarding funds if they seek to avoid reinforcing existing health inequalities. For example, an attractive grant application from a nearby location needs to be balanced against an assessment of need. Corporate foundations need to overcome what amounts to similarity bias due to corporate homophily. In addition, given that health inequalities are unfair, affect everyone, and are avoidable, and that interventions to reduce health inequalities are cost effective, both public and business policy should seek to reduce such inequalities (Woodward & Kawachi, 2000).

Our paper, seeking to determine how corporate foundations allocate their philanthropy, is organized as follows. First, we develop our hypotheses calling on theories from economic geography and corporate homophily. Second, we describe the methods and data we employ to examine health inequality and the distribution of health grants. Finally, we provide a discussion of what this means for corporate philanthropy and what changes may be needed for corporate philanthropy to reduce health inequality.

## Hypothesis Development

Traditionally corporate philanthropy has been considered a form of corporate social responsibility (CSR), which McWilliams and Siegel (2001) define as the private provision of public goods and which managers undertake if its benefits are greater than the costs.^1^ Most research is confined to the financial impacts of CSR within the firm and fails to examine how those public goods are distributed (Barnett et al., 2020). So, it is necessary to look beyond a simple economic cost–benefit analysis of firm benefits to explain the distributional consequences of CSR initiatives, such as philanthropy by examining the regional preference(s) that foundations reveal when awarding CSR health-related philanthropic grants.

The resources available to firms to invest in CSR are scarce. The resource allocation approach to CSR (e.g., McWilliams & Siegel, 2001; Waddock & Graves, 1997) suggests that when firms allocate CSR resources, including those by their philanthropic foundations, they tend to target CSR and philanthropic activities that ‘fit’ the firm given its mission, size, and position in the industry (Burke & Logsdon, 1996). Mackey et al. (2007) suggest that some forms of CSR may be substitutes for each other. In such cases, firms will allocate resources based on their preferences and/or, they argue, investor preferences (e.g., environmental CSR vs. employee CSR). If it were up to managers (with or without the preference of equity investors), then several frameworks exist to guide how their attention/resources should be allocated among different stakeholders based on such variables as power, influence, legitimacy, interests, and proximity (Driscoll & Starik, 2004; Mendelow, 1981; Mitchell et al., 1997).

In this paper, we hold the type of CSR constant by highlighting health-related philanthropy in order to focus specifically on preferences revealed by specific allocations based on geography. Hence within the framework of resource allocation for CSR, we argue that homophily and proximity are particularly relevant considerations influencing resource allocation decisions. Mackey et al. (2007) suggest that this allocation is determined by the market and industry. However, given limited CSR resources, another critical preference is revealed by which geographical areas receive grants from corporate foundations.

### Homophily

One of the most common explanations for inequitable distribution is based on homophily, which is the principle that “contact between similar people occurs at a higher rate than among dissimilar people” (McPherson et al., 2001, p. 416). Homophily pervades social life. As Kossinets and Watts (2009, p. 405) explain: “Friends, spouses, romantic partners, co-workers, colleagues, and other professional and recreational associates all tend to be more similar to each other than randomly chosen members of the same population with respect to a variety of dimensions, including race, age, gender, socioeconomic status, and education.” This principle often lies behind the inequitable distribution of health outcomes because unhealthy individuals have fewer contacts with healthy individuals and thus access to healthy role models and information about adopting health innovations, thus reinforcing poor health outcomes among the unhealthy (Centola, 2011).

Yet equally disconcerting is the role that homophily may play in influencing grantmakers in corporate foundations, resulting in allocative failures, which refer to a situation where “the outcomes of the social network [of foundations and beneficiaries] work against the ultimate goals of the network itself through a structure that reproduces homophily in resource allocation” (Kallman, 2017, p. 754). In the case of organizations, organizational homophily suggests that people in corporations and corporate foundations will be most attracted to potential beneficiary organizations that exhibit similar characteristics. For example, in a study of grant-making by US foundations to Chinese civil society organizations, Spires (2011, p. 306) found such organizational homophily in decisions by US foundations to direct funding toward “elite-led bureaucratic organizations controlled by the Chinese government and away from truly grassroots NGOs.”

In terms of grant-making, we expect that homophily will play a role insofar as corporate grant makers will be most interested in those proposals that best reflect the community norms used by the corporate foundations to determine what is an appropriate application (Marquis et al., 2007). Given homophily, grant proposals from areas which do not share the same community norms of professionalism, but may suffer from more severe health problems and greater needs due to the lack of effective interventions or medical resources, may not be winning competitive grants because they may not have access to the kinds of professional grant writers or other advice and resources needed to prepare proposals that are consistent with the professional norms in major metropolitan areas (Cantrell et al., 2008; Gautier & Pache, 2015). So unequal outcomes may be exacerbated by unequal capacity. Hence, we conclude that health grants by corporate foundations may reinforce existing health inequalities because they are not focused on the greatest need.

Homophily also shapes relational networks and thus the flow and diffusion of information. Within homophilous groups, the diffusion of information and opinions is quite rapid, but across groups, diffusion slows down (Golub & Jackson, 2012). This result suggests that the costs of information search are reduced by reference to others who exhibit homophily. Lower search costs provide an advantage to members of homophilous groups. Hence, homophily reduces information costs by focusing search on similar organizations. In addition, homophily constrains the flow of information and the network of contacts through which this information flows. Reduced costs and constraints thus lead to a tendency for corporate foundations to allocate greater CSR resources to homophilous beneficiaries. These factors will disadvantage potential recipients that are less similar, regardless of their manifest need for funding, resulting in a more inequitable distribution of health grants and, potentially, of health outcomes.

#### Hypothesis 1:

Counties with greater health needs receive less support from corporate foundations than counties with fewer health needs.

### Proximity

One of the most significant sources of homophily is based on geographic proximity (McPherson et al., 2001). “The most elementary proximity hypothesis is that interaction increases with geographic/physical propinquity. Being proximate is thought to encourage chance encounters and opportunities for interaction, which can lead to the formation of new relationships and the maintenance of existing ones” (Rivera et al., 2010, p. 105). Although Driscoll and Starik (2004) also saw proximity as an important dimension of stakeholder salience, they focused on proximity in the context of the natural environment physically located near the firm and its facilities. In terms of the allocation of philanthropic grants, we argue instead that increased proximity engenders homophily because it also reduces the effort or cost of information search (McPherson et al., 2001).

In addition to reducing the costs of information search, proximity also constrains the set of opportunities for interaction between actors—in this case, between corporate foundations and potential beneficiaries (Kleinbaum et al., 2013). These constraints shape the flow of information about opinions, norms, and values relevant to the awarding of grants. Proximity thus fosters the formation of community cultures surrounding philanthropy (Galaskiewicz, 1997; Marquis et al., 2007).

Abundant research in economic geography finds that geographic proximity or a “home bias” influences many kinds of business activities, including investment (Coval & Moskowitz, 1999; Ivkovic & Weisbenner, 2009) and CSR (Husted et al., 2016). Investors and corporations prefer to invest in their hometowns. In the case of corporate philanthropy, most philanthropy is directed to headquarter cities, which tend to be large metropolitan areas, and already have greater wealth, resources, and capabilities than non-metropolitan areas (Davis and Henderson, 2008). McElroy and Siegfried (1986) found that for 229 large companies located in 14 cities, most corporate contributions were focused on their headquarters’ city. In addition, there tends to be a high interrelationship between corporations and local NGOs, which are the recipients of corporate largesse (Galaskiewicz, 1997).

Hence, proximity reduces information costs by limiting search to nearby organizations. In addition, proximity also constrains the flow of information and the network of contacts through which this information flows. Reduced costs and constraints thus lead to a tendency for corporate foundations to prefer nearby beneficiaries, which often tend to be more homophilous. These factors will disadvantage recipients that are more distant, regardless of their manifest need for funding, resulting in a more inequitable distribution of health grants and potentially health outcomes.

#### Hypothesis 2:

*Counties that are home to the headquarters of corporations which have foundations receive greater support than those with no corporate headquarters.*

## Methods

We tested our hypotheses by combining datasets from the Robert Wood Johnson Foundation, which collects yearly county-level health data, and Candid (formerly Foundation Center) which records all grants made by corporate foundations on a yearly basis. By focusing on actual grants made, we observe the revealed preferences of the corporate foundations, rather than their stated preferences (Samuelson, 1948). Specifically, Candid collects data on corporate giving of U.S. companies through their respective foundations. The grant data are drawn from the IRS Form 990 s (Returns of Organization Exempt from Income Tax), which Corporate Foundations are required to report on an annual basis. These data only include cash disbursements from foundations to charities: nothing is mentioned or quantified in terms of in-kind donations or volunteer hours/efforts. Each observation from Candid is a specific grant made in a specific year from a corporate foundation. Alternatively, each observation is a specific grant received in a specific year by some charitable organization in a county. Candid records the donor’s name and location, recipient’s name and location (9-digit zip code), amount given and main activity area, which in our case is ‘health’. Using Census data, we are able to match the 9-digit recipient zip codes to 5-digit FIPS codes, which is used by the aforementioned Robert Wood Johnson Foundation data. As such, we are able to build a database at the county level, which links health needs to health-related corporate grants, along with other economic and socio-demographics variables. More specifically, we use data on corporate grants from Candid for the years 2009, 2013 and 2017 and measure county-level health needs just before these grants are made.^2^ Our approach is to look at corporate grants not from the grant maker’s end but rather from the recipient’s perspective. Our unit of analysis is, therefore, the panel county-year.

After accounting for missing variables for some counties (health needs in some years are unassessed), we have 3131 counties (out of 3144 US counties registered) for which we observe data for at least 2 years. The total number of observations is 8027. County-level health grants in any year $t$ are calculated by summing all health grants received by a county in year $t$. Candid categorizes each grant according to its proprietary Philanthropy Classification System (PCS), which is slightly more nuanced and detailed than a recipient’s National Taxonomy of Exempt Entities (NTEE) code. We adopt a conservative approach by limiting our analysis to health grants, and within health grants we also omit all grants that relate to medical schools, scholarships, publications, and research. Examples of non-research-related health grants include antidiscrimination in health-care access, burn care, cancers (care, social services), domestic violence shelters, emergency care, health insurance, mental health-care and counseling, obesity, prenatal care, school-based health-care, substance abuse prevention, women’s services, and youth development and organizing.

### Estimation Strategy

Our first hypothesis stipulates that counties with the greatest health needs are unlikely to receive health-related corporate grants, thereby reinforcing existing health inequalities. Our second hypothesis posits that counties with at least one headquarters of corporations with foundations receive more grants than those counties with no corporate headquarters. To test our hypotheses, we estimate the likelihood and the amount that counties receive in health-related corporate grants as a function of county socioeconomic and health needs, that is:

1 $$ Grants_{c,t} = \alpha + \beta \cdot Health Needs_{c,t - 2} + \mu HQ_{c} + \gamma Z_{c,t - 2} + \rho UrbRur_{c} + \varepsilon_{c,t} $$

We are seeking to estimate both the likelihood (binary) and amount (continuous) of grants that a county, c, receives in year $t$ contingent on its health needs in year $t-2$, whether the county has at least one HQ of any corporation with a foundation (to account for geographic proximity), and a vector of time-varying county-level controls (median household income, unemployment rate,% non-Hispanic African Americans,% Hispanic, Gini, Health-care costs per capita) denoted by Z, as well as a time-invariant control (Urban–Rural county categories). We proxy health needs by ‘access to health-care’, i.e., the total number of providers of primary and mental health-care (access to health providers), as well as the percentage of uninsured adults in each county.

We first estimate Eq. (1) by pooling the data and adding in year fixed effects to the estimating equation. To analyze whether certain characteristics make them more likely to receive grants, and to analyze the level of grants received by counties as a function of county attributes, we use the two-part model for non-count data developed by Belotti et al. (2015). Two-part models are very common in count data (e.g., Cameron & Trivedi, 2013) where researchers first seek to analyze the likelihood of say, rain, and subsequently want to analyze the number of days it rained in a period of time. In this case, researchers can use the Poisson model or the negative binomial model. As our data are non-count data, a two-part model for non-count data is required. Here, the first part is used to analyze the likelihood of a county receiving grants via a logit model, and the second part is used to analyze the value of grants received conditional on grants having been received. The second part is calculated using OLS, and we log-transform the dependent variable (grants per capita) for ease of interpretation and because the grants data are highly dispersed. With the pooled data analysis, we cluster the standard errors at the county level.

Secondly, we estimate Eq. (1) using a population-average logistic estimator as well as a between-effects panel regression. We use population-average and between-effects because we are interested in the differences between counties, rather than the effect of changes within the county over time. For the span of years of our analysis, changes within the county are expected to be minimal. With these two panel data methods, we calculate standard errors using the bootstrap method.

### Variable Definitions

*Health Needs* The Robert Wood Johnson Foundation has amassed data on health-related variables across US counties since 2010. These data are sourced from several agencies in the US. We measure health needs with two measures. First, we create an index that sums the number of primary-care physicians and mental health providers per 100,000 people for each county. We call this access to health providers. Second, we use the percentage of uninsured adults in each county. The Robert Wood Foundations lumps these measures into what it calls ‘access to care’. Higher access to care implies lower health needs in a county and vice-versa. The data on primary-care physicians are sourced from the American Medical Association, whereas the data on mental health providers are sourced from the Centers for Medicare and Medicaid National Provider Identification. The percentage of uninsured adults in a county comes from the Small Area Health Insurance Estimates.

*Corporate Health Grants* Corporate health grants are used as the dependent variable. In the first instance, we compare recipient counties with non-recipient counties and test whether county attributes, including health needs, affect the likelihood of receiving grants. In the second instance, we look at the value of grants per head received by a county as the dependent variable.

*Additional health and demographic data* As control variables, we use measures obtained from the Robert Wood Johnson Foundation data. First, we include in our estimation health-care costs per county per year (McCullough & Leider, 2016), which measure Medicare costs at the county-year level. Such data, sourced from the Dartmouth Atlas of Health-Care, act as a control since counties spend differentially on health-care for a variety of reasons and may therefore affect health outcomes. To control for demographic factors (McLaughlin and Stokes, 2002), we use (log of) median household income which is sourced from the Small Area Income and Poverty Estimates (SAIPE) and the unemployment rate which we source from the St. Louis Federal Reserve, whereas population,% of non-Hispanic African Americans,% Hispanic are obtained from other US Census Estimates. To account for income disparities within a county, we include county-level Gini coefficients, which we directly source from SAIPE (Shi et al., 2005). Further, we use the National Center for Health Statistics (NCHS) data to categorize counties into one of the following: (a) large central metro, (b) large fringe metro, (c) medium metro, (d) small metro, (e) micropolitan, (f) non-core.

## Results

### Summary Statistics

**Fig. 1:**
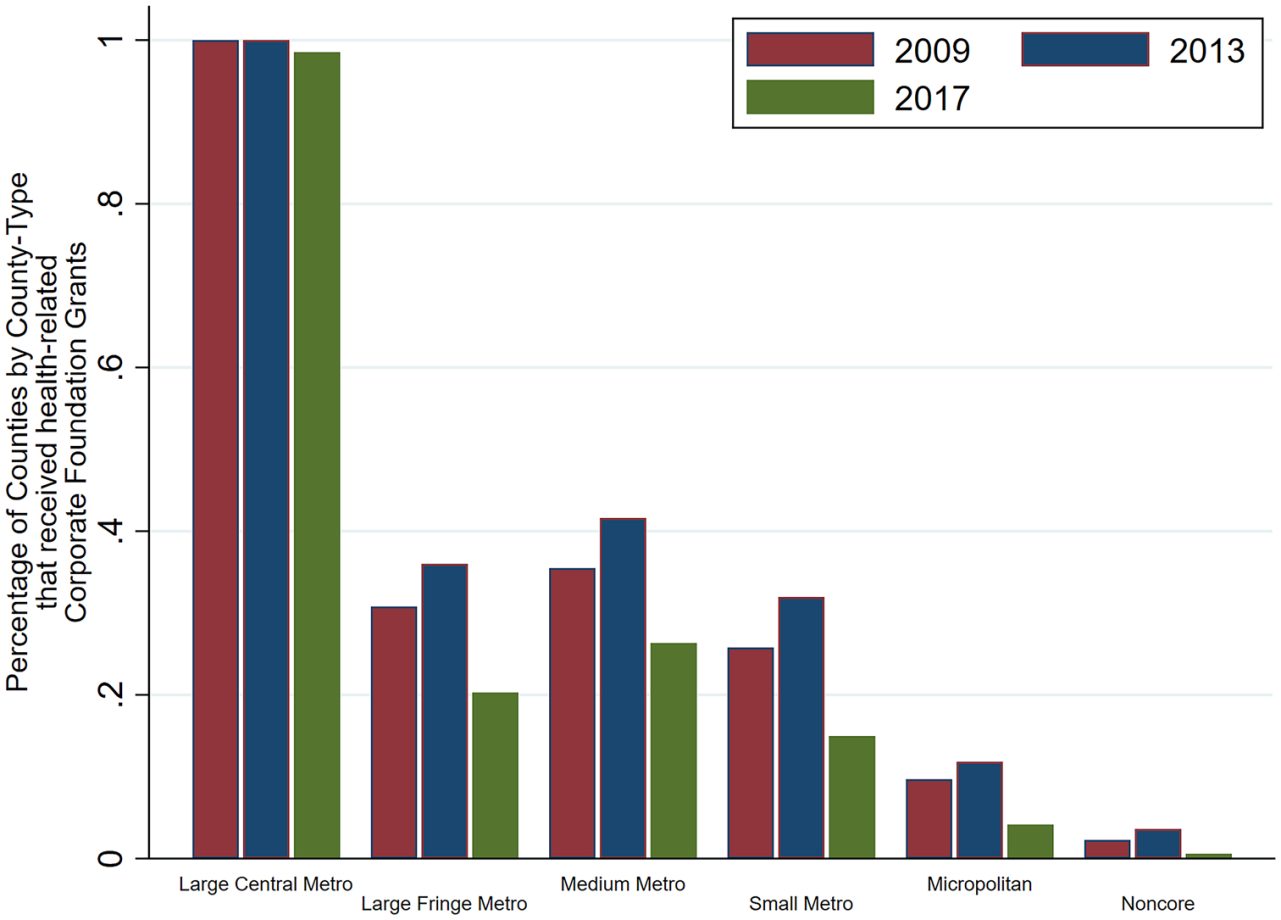
Proportion of counties per the urban–rural classification, that received health grants

**Fig. 2:**
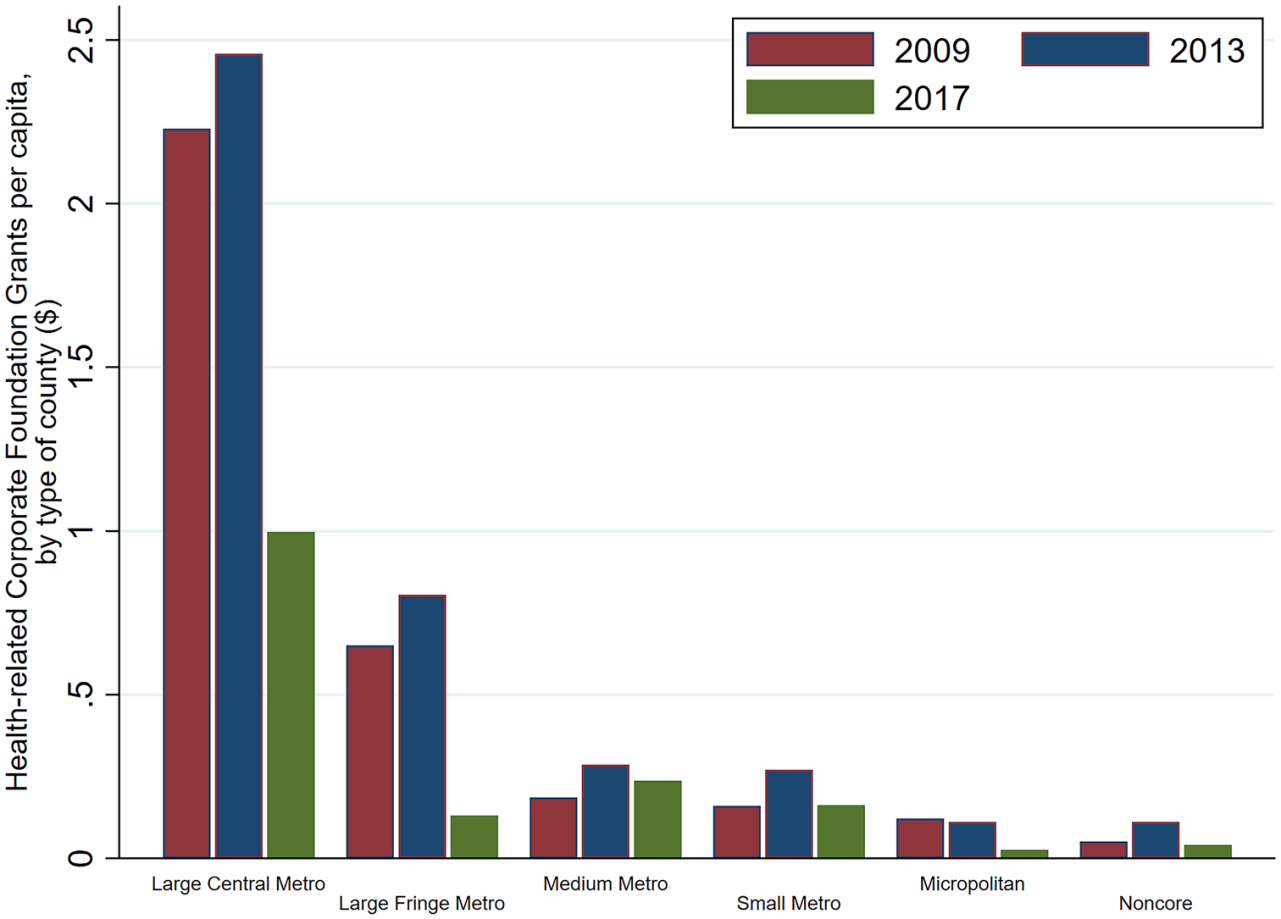
Grants per head by type of county

If we look at the distribution of counties per the urban–rural classification receiving health grants, Fig. 1 shows that almost 100% of all large central metropolitans received some health-related grants from corporate foundations. Around 20–40% of large fringe metros, medium metros, and small metros received some grants, and the numbers are lower for micropolitans and non-core areas. Over the 3 years of analysis, some trends are worth mentioning. First, our data from Candid shows that 2013 was the year where more (health) grants were made. In 2017, the number of corporate foundation (health) grants fell quite dramatically, and the major losers of this overall decline were not large central metros. Rather, from 2013 to 2017, the proportion of recipient counties in each of the groups characterized as large fringe metros, medium metros and small metros dropped by almost 20 percentage points. Examining the average value of health grants (including zero) per capita by type of county, our calculations show, in Fig. 2, that large metropolitan counties receive a significantly higher amount than all other types of counties. In 2013, for example, on average large central metros received $2.50 per head in health grants from corporate foundations. This figure drops to under $1.00 for large fringe metropolitans, while medium, small metros receive approximately $0.25 per head, while micropolitans, and non-core counties receive even lower amounts per head. Again, there is a time trend over the years. In terms of value, even large central metros received less in 2017 compared to 2013. Health grants per capita dropped from $2.50 per head to just over $1.00 per head.

To compare time trends across counties and across time, Table 1 presents summary statistics at the county-year level. Across all counties and all three years, the average number of health providers per 100,000 is 22.8. The average percentage of uninsured adults is around 17%. On average, only 16.3% of all US counties received a health grant across our years of analysis. The summary statistics show that there are both within- and between- county variations in the data. Overall, across all variables, we note that the largest sources of variation in the data arise between counties rather than within counties, except for access to health-care providers. With regards to the dependent variable, there is high variation in corporate grants. Most counties receive zero grants, while some receive a significant amount per capita. We now examine the non-zero grants.

**Table 1:** Summary statistics at County-year level

	Mean	Std. Dev	Min	Max
Access to health-care providers (# per 100,000 people)				
Overall	22.819	40.104	0.000	604.894
Between		18.471	0.000	302.450
Within		36.373	− 279.626	400.016
% Uninsured				
Overall	0.167	0.057	0.027	0.428
Between		0.055	0.032	0.428
Within		0.022	0.076	0.259
Health grants per capita ($)				
Overall	0.211	2.726	0.000	184.612
Between		2.022	0.000	103.251
Within		1.638	− 92.596	81.572
At least one corporate HQ in county (dummy variable)				
Overall	0.037	0.189	0.000	1.000
Between		0.175	0.000	1.000
Within		0.000	0.037	0.037
Counties that received grants (proportion)				
Overall	0.163	0.369	0.000	1.000
Between		0.300	0.000	1.000
Within		0.188	− 0.504	0.829
% Non-hispanic African Americans				
Overall	0.090	0.139	0.000	0.863
Between		0.143	0.000	0.854
Within		0.005	0.025	0.179
% Hispanics				
Overall	0.084	0.126	0.000	0.972
Between		0.132	0.001	0.962
Within		0.008	− 0.041	0.209
Log of median household income				
Overall	10.714	0.241	9.862	11.743
Between		0.234	9.895	11.686
Within		0.059	10.405	11.023
Unemployment rate (%)				
Overall	7.084	3.177	1.242	27.325
Between		2.365	2.064	23.967
Within		2.264	− 1.945	16.113
Gini				
Overall	0.441	0.035	0.332	0.599
Between		0.033	0.344	0.598
Within		0.013	0.327	0.549
Large central metro				
Overall	0.0254	0.157	0.000	1.000
Between		0.145	0.000	1.000
Within		0.000	0.0254	0.0254
Large fringe metro				
Overall	0.130	0.337	0.000	1.000
Between		0.322	0.000	1.000
Within		0.000	0.130	0.130
Medium metro				
Overall	0.131	0.337	0.000	1.000
Between		0.324	0.000	1.000
Within		0.000	0.131	0.131
Small metro				
Overall	0.122	0.328	0.000	1.000
Between		0.318	0.000	1.000
Within		0.000	0.122	0.122
Micropolitan				
Overall	0.221	0.415	0.000	1.000
Between		0.403	0.000	1.000
Within		0.000	0.221	0.221
Noncore				
Overall	0.370	0.483	0.000	1.000
Between		0.494	0.000	1.000
Within		0.000	0.370	0.370

Number of counties: 3131; number of observations: 8027

**Fig. 3:**
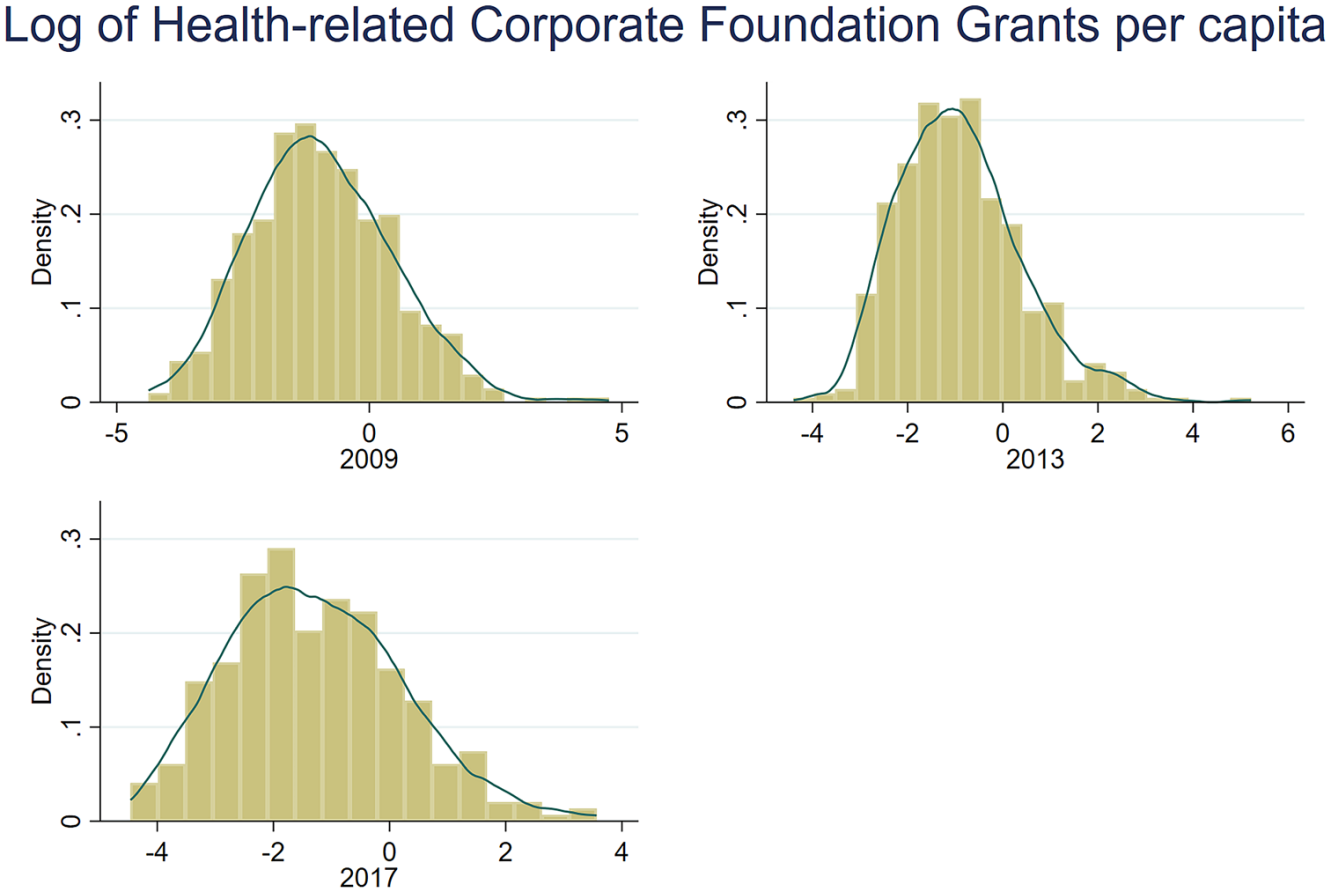
Distribution of the log of (non-zero) health grants

Figure 3 shows the distribution of the log of non-zero grants for each of the three years (same *y*-axis scale). The data show that most counties receive less than $1.00 per head (log of 1 = 0), and the distribution is slightly right skewed. The distributions of non-zero grants across each of the three years suggest that there is not much difference between the three years.

### Likelihood of Receiving Grants and Amount of Grants Received Based on Health Status

To test hypothesis 1 with pooled data, we run the aforementioned two-part model on Eq. (1) with standard errors clustered at the county level. Table 2 shows these results. The regressions show coefficients (not odds ratio) in two columns: the logistic first part results, and the OLS second part results. These results confirm a few stark findings. Counties with higher health needs (i.e., lower access to health providers and a higher percentage of uninsured adults) are less likely to be recipients of foundation health grants. Our pooled logistic regressions suggest that as the number of providers goes down by 10 for every 100,000 people, the odds of receiving health grants goes down by 12%.^3^ Similarly, as the rate of uninsured adults goes up, the odds of receiving health grants go down. For every one percentage point increase in the rate of uninsured adults, the odds of receiving health grants goes down by 9%. Richer counties denoted by higher median household incomes are more likely to be recipients of health grants. However, as unemployment goes up, the likelihood of receiving grants goes up. We also find that more unequal counties, which are those with higher Gini coefficients, are more likely to receive grants. A 0.01 increase in Gini is accompanied with a 20% increase in the odds of receiving grants. In terms of urban–rural classifications (not shown in the table for parsimony), larger counties are progressively more likely to receive grants than smaller ones.

**Table 2:** Two-part model with likelihood and amount of Health Grants per capita to counties

	Pooled data		Panel methods	
	Logit	OLS (with log)	PA logit	BE (with log)
Access to health-care providers (# providers per 100,000 people)	0.0121^***^	0.00206^+^	0.00451^***^	0.00197
	(0.00237)	− 0.00106	(0.00101)	(0.00125)
% Uninsured	− 9.577^***^	− 2.183	− 3.598^**^	− 2.496^+^
	(1.333)	1.386	(1.102)	(1.437)
At least one corporate HQ in county	2.286^***^	0.388^**^	2.439^***^	0.364^*^
	(0.250)	0.144	(0.206)	(0.163)
% Non-hispanic African American	0.882^*^	1.090^*^	− 0.135	0.757
	(0.417)	(0.483)	(0.513)	(0.470)
% Hispanic	3.450^***^	0.390	1.879^***^	0.246
	(0.415)	(0.422)	(0.379)	(0.503)
Median household income	2.348^***^	0.415	2.379^***^	− 0.117
	(0.331)	(0.328)	(0.314)	(0.386)
Gini	18.881^***^	5.661^**^	18.09^***^	3.863
	(1.761)	(1.744)	(1.556)	(1.579)
Unemployment rate	0.0912^***^	− 0.0558^**^	0.136^***^	− 0.0624^***^
	(0.0222)	(0.0182)	(0.0157)	(0.0177)
Urban–rural County classifications	YES	YES	YES	YES
Year fixed effects	YES	YES	–	–
Observations	8027	1307	8027	1307
Wald chi-2	919.76	–	1254.67	309.74
Log (pseudo)likelihood	− 2162.743	− 2155.614		
Pseudo/adjusted R-squared	0.394	0.179	–	0.235

Standard errors clustered at county in parentheses for pooled OLS, bootstrapped std. errors for panel models

^+^*p* < 0.1, *p < 0.05, **p < 0.01, ***p < 0.001

Turning to the OLS regression, we note similar results in terms of access to health-care providers, but insignificant results (due to the clustering of standard errors at the county level) with regard to the percentage of adults uninsured. Conditional on receiving grants, a decrease of 10 health providers for every 100,000 people is accompanied by a reduction of 2.1% in the value of health grants. In a nutshell, counties with greater health needs are less likely to receive grants, confirming Hypothesis 1 that counties with fewer health resources receive less support through corporate health grants than counties with higher levels of health resources. The positive trend we observed with unemployment in the logit model reverses such that counties with higher unemployment are more likely to receive grants, but such grants are lower in value than counties with lower levels of unemployment.

Where then do foundations issue health grants? Hypothesis 2, which states that the presence of a corporate HQ in a county increases the likelihood of receiving a health-related grant, is strongly supported. The odds of receiving a health grant goes up by more than 9 times when a county houses the HQ of at least one company which has a grant-making foundation. The value of grants is also higher for counties that are home to at least one corporate HQ. Counties that have at least one corporate HQ receive on average 47% more in health grants per capita^4^ than counties in which there is no corporate HQ. Moreover, large central metropolitans are more likely to receive grants compared to other county types. Proximity to corporate HQ attracts more grants. Hypothesis 2 is supported.

Our results are also supported using panel methods which are estimated with bootstrapped standard errors. In particular, we use *xtlogit* (i.e., generalized linear model with logit link and binomial family) in Stata with population averages, and we use between-effects *xtreg*. The panel methods estimates are generally smaller than the pooled OLS estimates. However, the statistical significance does not change. Under the panel estimations, as the number of providers goes down by 10 for every 100,000 people, the odds of receiving health grants goes down by 4.5%. The relation between health providers and grants per capita is not statistically significant anymore. With regards to the percentage of adults uninsured, a 1 percentage point increase in the proportion of uninsured adults is associated with a 3.5% reduction in the odds of receiving a grant.^5^ Similarly, a 1 percentage point increase in the proportion of uninsured adults is associated with 2.47% lower value of health grants per capita. The results for the presence of a corporate HQ in the county are similar to the ones reported in the pooled models.

### Robustness Checks

As a robustness check, in terms of methodology, we ran the same logistic model as before along with a tobit model. This combination of a logit/probit (binary outcome) with a tobit model (continuous outcome) is common practice in public policy literature. Specifically, these combinations are used to assess factors that determine grants received by municipalities (Dahlberg & Johansson, 2002) and local governments (Dubois & Fattore, 2011). Similar to government grant research, our data is zero-inflated with a majority of counties not receiving any grants. As such, we can say that the data is left-censored at zero. Results from these two regressions confirm exactly what we observed in our two-part model regressions.

A further concern with our paper is that perhaps our focused look at (non-research) health-related grants is too narrow. On the one hand, this focus allows us to be more precise in establishing a more direct link between corporate philanthropy and health-care needs. On the other hand, it is plausible that grants in education, public safety, social sciences, arts and culture, and other areas along with health are all related to health needs. Using a more holistic approach, perhaps community development and education grants can alleviate inequality in health. We, therefore, run the same tests as reported earlier but this time with total corporate grants rather than health-related corporate grants. We still exclude all grants whose description contains one or more of the words “research”, “publications”, “scholarships”. Considering all donations, 1583 counties received some grant for a total of 3333 county-year observations. Results are shown in Table 3.

**Table 3:** Robustness checks using all grants

	Panel methods	
	PA logit	BE (with log)
Access to health-care providers (# providers per 100,000 people)	0.00420***	0.00621***
	(0.000657)	(0.00123)
% Uninsured	− 1.279^+^	− 2.695**
	(0.769)	(0.870)
At least one corporate HQ in county	3.052***	1.116***
	(0.598)	(0.161)
% Non-hispanic African American	− 0.270	0.875***
	(0.296)	(0.282)
% Hispanic	0.415	0.646^+^
	(0.361)	(0.350)
Median household income	2.051***	0.115
	(0.187)	(0.239)
Unemployment rate	0.110***	− 0.0747***
	(0.0113)	(0.0126)
Gini	10.509***	7.136***
	(1.035)	(1.182)
Urban–rural County classifications	YES	YES
Observations	8027	3333
Wald chi(2)	1336.98	873.82
Adjusted R-squared	–	0.274

Bootstrapped standard errors in parentheses, ^+^*p* < 0.1, *p < 0.05, **p < 0.01, ***p < 0.001

Regarding Hypothesis 1, we find that on average a decrease of 10 health providers per 100,000 people is associated with a 4.2% lower likelihood of receiving some corporate donation. The OLS results show that, contingent on receiving grants, a decrease of 10 health providers per 100,000 people is accompanied by a per capita reduction of 6.2% in the value of total corporate grants received by a county. At the same time, a 1 percentage point increase in the number of uninsured adults is accompanied by a reduction of 1.3% in the odds of receiving corporate grants, while the same 1 percentage point increase is associated with a 2.66% reduction in the value of grants received per capita. Overall, we can argue the same as we did before but with a bigger punch. Corporate grants in general are not going to the neediest counties.

With regards to Hypothesis 2, as expected, our results are even stronger when all corporate grants are considered. If a county has at least one corporate HQ, its odds of receiving any kind of corporate grant goes up by 21 times, whereas the value of grants it receives goes up by 205% per capita.

## Discussion and Conclusion

The COVID-19 pandemic has exposed health inequalities in the United States. We would expect corporate philanthropy to provide for the poor and disadvantaged by addressing the root causes of these inequalities, not reinforce them. In this paper, we hypothesized and found that health-care grants by corporate foundations predominate in areas with lower health-care needs, such that counties which have fewer uninsured citizens and where citizens have greater access to primary-care physicians and mental health providers, are the more likely recipients of health-related corporate grants. Further, even among the “winners”, more health grants are awarded to counties with less severe health needs. There is a strong association between counties that receive health grants and counties where the foundation’s corporate headquarters is located.

### Theoretical Implications

We find evidence that two factors are associated with the severity of health problems. First, corporate giving is generally focused on the local community, which for corporate foundations tends to be large metropolitan areas, and which has the consequence of reinforcing the “rural–urban health divide” (Miller & Vasan, 2021). Our finding that the odds of receiving a health grant goes up by more than 9 times when a county houses a HQ of at least one company that has a grant-making foundation suggests that proximity matters. Second, corporate foundations are more likely to fund grant applications emanating from well-resourced counties and NGOs. The implication of both tendencies is that corporate health philanthropy tends to reinforce pre-existing health inequalities and even exacerbate them.

Although these findings do not conclusively establish causality due to the lack of randomized trials, they strongly suggest that corporate philanthropy is inadvertently worsening inequities in the distribution of health grants in rural vs. urban counties in the United States. Instead of assuming that CSR and/or philanthropy are inherently good, the social impacts need to be established independently, rather than be assumed. This opens up an entirely new question for CSR research: Under what conditions does CSR generate positive social impacts? This question is consistent with the pragmatic approach recommended by (Margolis & Walsh, 2003), when they suggested that research should be reoriented to investigate the social impacts of corporate practices like CSR and then determine how these practices could be improved to increase social impact. Sadly, almost twenty years after (Margolis & Walsh, 2003), the literature fails to address the fundamental question of whether CSR or philanthropy actually generate positive social impact (Barnett et al., 2020). Given the strong evidence provided in this paper that health grants by corporate foundations appear to increase inequity, the literature must move forward to study the impacts of social initiatives and develop theory to explain when such initiatives will have positive impacts and when not.

### Normative Implications

From several normative perspectives, this distribution of health grants seems unfair. If we look at Rawls’ well-known difference principle of justice, it reads: “Social and economic inequalities are to be arranged so that they are both: (a) open to all under conditions of fair equality of opportunity, and (b) to the greatest benefit of the least advantaged…” (Rawls, 1971, p. 266). Caring for the least advantaged should be a priority in policy about the distribution of health resources and would require corporate foundations to direct grants to counties with the greatest needs. Moving beyond Rawls’ focus on the distribution of advantages, Sen (2008) notes that Rawls does not take into account differences in the ability of people to turn these advantages into a good life. Working within this “capability approach,” Nussbaum (2011) specifically includes good health as a capability that a political order must ensure for human dignity. If having good health is a basic requirement for a just society, corporate foundations should align their giving so that those with greater health needs are able to obtain good health.

### Practical Implications

Unwittingly, corporate foundations may be complicit in worsening the health resource gap between rural, small, poor counties and more wealthy, large, urban counties. Instead of going to places where the need is greatest, funding appears to go to places where the need is less, but which are closer or more similar to home. Grant recipients located in the same county as a large corporate headquarters would benefit from this location bias. But if corporate foundations want to achieve the greatest benefit from their donation dollar, they should change the criteria for awarding funding. Lantz (2019, p. 38) argues that there are “institutional, systemic, and public policy drivers of population health problems and distributional disparities” which need to be considered.

Clearly an assessment of the severity of health needs should be at the center of the decision criteria for awarding health grants. Corporate foundations need to overcome what might amount to a “similarity bias.” Not only are health inequalities unfair, affect everyone, and avoidable, but health interventions to reduce health inequalities are cost effective (Woodward & Kawachi, 2000). To put the “love of humanity” back in corporate philanthropy, corporate foundations should become aware of the implicit bias that might be driving their funding decisions and actively work to reduce inequalities in health grants.

Philanthropist MacKenzie Scott modeled such a needs-based approach in her recent donation of over four billion dollars to non-profits and community organizations throughout the United States (Scott, 2020). To assist her in the distribution of these donations, she assembled a panel of experts who “took a data-driven approach to identifying organizations with strong leadership teams and results, with special attention to those operating in communities facing high projected food insecurity, high measures of racial inequity, high local poverty rates, and low access to philanthropic capital” (Scott, 2020). She donated to universities, but not the usual suspects like Harvard University or Stanford, but Navajo Technical University and Texas A&M International University. Essentially, she took the similarity bias seriously and maintained a highly focused effort to donate to organizations in all 50 states, D.C. and Puerto Rico based on need and potential impact.

### Limitations and Future Research

Notwithstanding the above analysis, this paper is not without limitations. First, although we find that physical distance from corporate foundations increases health inequality, the COVID-19 example cited in our introduction regarding the two hospitals suggests that there may also be cultural distance between donors and recipients that needs to be examined. A deeper look at the geographical, cultural, and ideological distance between donor and recipient is needed. Second, grants made through corporate foundations are not the only grants received by US counties. Some counties may be receiving direct support from private companies, support from foundations sponsored by philanthropists, and other forms of support. Such data are difficult to source but could support or reject our claims about homophily. Third, this paper has not accounted for the particular set of micro-level mechanisms that could explain why grants are awarded to certain recipients. Fourth, homophily is not constant over time. Homophily has explained why people are attracted to similar others, but we do not account for how homophily varies over time. Failure to account for variation in homophily is a limitation, but a critical question as it would help provide solutions to the problems it generates.

These limitations, in our opinion, should be the work of further research. To our knowledge, ours is the first paper to empirically assess the unequal distribution of corporate grants. Our results should provide grounds for future research. For example, one implication of our research is that the “marginal benefit” of corporate giving on health outcomes may be negative if it exacerbates the inequality of health resources. This effect would need to be studied in a separate paper, given the challenges of detecting health outcomes at the county level based on foundation grants to organizations within the county. In addition, our analysis treats homophily as a constant, but future research needs to explore what factors enable individuals and organizations to counteract the tendency to be attracted to similar others. An understanding of these factors would require examining the cognitive processes of decisionmakers and would require data beyond the type and amount of grants made. Finally, our entire analysis was based on health. While this is a very important area, possibly the most important aspect of human welfare, other dimensions such as income inequality and education deserve attention as well.

## Footnotes

1 In the case of philanthropy, an organization will donate funds if they believe the benefits, both monetary and non-monetary (e.g., reputational and intangible benefits) are greater than the opportunity cost of using those funds for the next best purpose.

2 Due to data availability, for grants made in 2009, health needs are assessed in 2006; for 2013, needs are assessed as an average of values in 2010–2011; for 2017, needs are assessed as an average of values in 2014–2016. Economic and Socio-demographics data are collected two years prior to each year of grants. For sake of parsimony, the models in this paper will denote independent variables at $t-2$ for every year in which we observe health grants. The Robert Wood Johnson Foundation sources these data from various agencies in the US, some of which are mentioned in this paper.

3 Calculated as (*e*^*coef.*^ − 1) * 100%

4 Since the second part of the two-part model uses the log transformation of the dependent variable (health grants per capita), the interpretation of the coefficient is (*e*^coef^ − 1)*100%.

5 Because % uninsured is measured on a scale of [0,1], change in odds ratio is ${(e}^{-0.03598}-1)$.
